# IFNγ-secreting T cells that highly express IL-2 potently inhibit the growth of intracellular *M. tuberculosis* in macrophages

**DOI:** 10.3389/fimmu.2024.1469118

**Published:** 2024-11-07

**Authors:** Liying Zhu, Bo Wang, Jin Gu, Jiayu Zhou, Yuan Wu, Wei Xu, Min Yang, Xia Cai, Hongbo Shen, Lu Lu, Feifei Wang

**Affiliations:** ^1^ Shanghai Institute of Infectious Disease and Biosecurity and Key Laboratory of Medical Molecular Virology (MOE/NHC/CAMS), Biosafety Level 3 Laboratory, Department of Medical Microbiology and Parasitology, School of Basic Medical Sciences, Shanghai Medical College, Fudan University, Shanghai, China; ^2^ Guangdong Provincial Key Laboratory of Major Obstetric Diseases, The Third Affiliated Hospital of Guangzhou Medical University, Guangzhou, China; ^3^ Shanghai Clinical Research Center for Infectious Disease (tuberculosis), Shanghai Key Laboratory of Tuberculosis, Shanghai Pulmonary Hospital, Institute for Advanced Study, Tongji University School of Medicine, Shanghai, China; ^4^ Shanghai Sci-Tech Inno Center for Infection & Immunity, Shanghai, China

**Keywords:** IFNγ-secreting T cells, *Mycobacterium tuberculosis*, BCG, IL-2, intracellular mycobacteria, mRNA sequencing

## Abstract

Cytokine of interferon-gamma (IFNγ) plays a vital role in the immune response against *Mycobacteria tuberculosis* (Mtb) infection, yet the specific function of T cells producing IFNγ in this process remains unclear. In this study, we first isolated IFNγ^+^CD3^+^ T cells induced by Mtb antigens using surface staining assays. which showed a strong ability to inhibit the growth of intracellular mycobacteria in macrophages. Peripheral blood mononuclear cells (PBMCs) from healthy individuals were then challenged with Bacillus Calmette–Guérin (BCG) or Mtb, respectively, to sort IFNγ-secreting T cells for mRNA sequencing to analyze the gene expression patterns. The results of the integrated data analysis revealed distinct patterns of gene expression between IFNγ^+^CD3^+^ T cells induced by the BCG vaccine and those induced by Mtb pathogens. Further, unlike Mtb-induced cells, BCG-induced IFNγ^+^CD3^+^ T cells expressed high levels of interleukin-2 (IL-2), which increased the frequencies of these cells and the production of effector cytokines IFNγ and IL-2. Our findings suggested that IFNγ^+^CD3^+^ T cells with high IL-2 expression presented potent effector functions to inhibit intracellular Mtb growth, while Mtb infection impaired IL-2 expression in IFNγ^+^CD3^+^ T cells.

## 1 Introduction

Tuberculosis (TB), caused by *Mycobacterium tuberculosis* (Mtb), is the second leading cause of death from a single infectious agent, surpassed only by COVID-19 (1, 2). The global control of TB, especially multidrug-resistant TB (MDR-TB), is hindered by the lack of effective vaccines and treatments, including powerful immunotherapies or host-directed therapies (HDT). Developing effective TB vaccines or HDTs necessitates a deeper understanding of protective anti-TB immunity (3).

Mtb, an airborne intracellular pathogen, primarily infects alveolar macrophages. T cells are crucial in the host immune response, controlling Mtb replication and limiting disease progression and recurrence (4, 5). Such as in human immunodeficiency virus (HIV)-infected individuals, TB co-infection is the leading cause of death attributing to the loss or destruction of CD4 T cells function. Thus, it is essential to further understand the protective immune response of T cells against Mtb. And, it is proved that many types of effector T cells, such as Th1-type CD4 T cells, CD8 cytotoxic T lymphocytes (CTLs), and unconventional T cells such as γδ T cells, MAIT cells, and natural killer T cells are all vital for host protective immunity against Mtb through different mechanisms (6, 7).

Cytokines significantly regulate T cell responses to Mtb infection, exerting substantial immunomodulatory and immunostimulatory effects (8). Interferon-gamma (IFNγ) is particularly important in TB immunity (9), with its significance established in individuals with genetic defects in IFNγ (10, 11). IFNγ, along with tumor necrosis factor (TNF)α and interleukin-2 (IL-2), constitutes the CD4 Th1 cytokines involved in the Th1 immune response. Th1 cells producing IFNγ, TNF-α, and IL-2 are multifunctional CD4 T cells (12, 13). Additionally, CD8 effector T cells can produce IFNγ, which activates macrophages to kill intracellular bacteria (14, 15). However, the precise impact of IFNγ-secreting T cells on the overall anti-TB immune response remains unclear.

IFNγ expression can be induced by both Mtb pathogens and the Bacillus Calmette-Guérin (BCG) vaccine. The roles of IFNγ-secreting T cells induced by different stimuli in the anti-TB immune response needs further investigation. The BCG vaccine, the only authorized TB vaccine, is effective in protecting against severe TB in infancy and early childhood (16–18). Previous research indicates that intravenous administration of BCG in *Macaca mulatta* enhances antigen-specific CD4 and CD8 T cell responses, boosting protection against Mtb challenge (19). However, the immune responses elicited by BCG differ from those induced by Mtb. Recent studies suggest that systemic BCG administration confers protective trained immunity against Mtb by reprogramming hematopoietic stem cells (HSCs) in the bone marrow via the IFN-II response, whereas Mtb reprograms HSCs through an IFN-I response, impairing protective trained immunity by inhibiting myelopoiesis (20). Therefore, exploring the differences between the immune reactions elicited by the BCG vaccine and Mtb pathogens is crucial.

In this study, we hypothesized that that IFNγ-secreting T cells induced by the BCG vaccine and Mtb pathogen exhibit unique effector roles. Our research results addressed this hypothesis and found that antigen-stimulated IFNγ-secreting T cells significantly inhibit the growth of intracellular mycobacteria in macrophages. Transcriptome analysis of IFNγ-secreting T cells revealed that the gene expression patterns of T cells derived from BCG stimulated healthy human peripheral blood mononuclear cells (PBMCs) were different from those stimulated by Mtb. Our findings also showed that IFNγ^+^CD3^+^ T cells induced by BCG and antigens highly express IL-2, while IL-2 expression unaltered by Mtb pathogen stimulation and diminished in TB patients.

## 2 Materials and methods

### 2.1 Human subjects

This study was approved by the Institutional Review Board and Biosafety Committee at Shanghai Pulmonary Hospital (SPH), Shanghai, China. Written informed consent was obtained from all adult participants (≥18 years). We enrolled individuals with active TB and healthy controls (HC), excluding those with infections (HBV, HCV, HIV), other infectious diseases, or cancers. TB patients were confirmed by routine tuberculosis diagnostic tests, including the tuberculin purified protein derivative (PPD) test and, when necessary, the QuantiFERON^®^ TB test. Whole blood samples were collected from enrolled subjects. (21–23).

### 2.2 PBMC and IFNγ^+^CD3^+^ T cell isolation

Human peripheral blood mononuclear cells (PBMCs) from 3 healthy donors were isolated from participants using Ficoll-Paque PLUS medium (Cytiva, USA) and cultured in RPMI 1640 medium with supplements (23–25). PBMCs from healthy donors or TB patients were stimulated with Mtb antigen [(E)-4-hydroxy-3-methyl-but-2-enyl pyrophosphate (HMBPP, Sigma-Aldrich, 95098) plus purified protein derivative (PPD)], BCG, or H37Rv for 6 hours. CD3 T cells were isolated using negative selection (Miltenyi, Germany, 130-096-535), and IFNγ^+^ cells were enriched using an IFNγ cell enrichment and detection kit (Miltenyi, Germany, 130-054-201).

### 2.3 Mycobacteria culture and infection of host cells

*Mycobacterium bovis* Bacillus Calmette–Guérin (BCG, ATCC 35733) and Mtb H37Rv (ATCC 27294) were grown into log phase at 37°C in Difco Middlebrook 7H9 broth medium (Becton Dickinson, 271310) with 10% oleic acid-albumin-dextrose-catalase (OADC) Enrichment (Becton Dickinson, 212351), 0.05% (v/v) Tween 80 and 0.2% (v/v) glycerol as our previously described (22).

For differentiation of human monocytes-derived macrophages (hMDMs), CD14 cells were enriched by isolation using the MASC CD14 Microbead kit (Miltenyi, Germany, 130-050-201) and cultured under the medium containing RPMI1640, supplemented with L-glutamine (2 mM), sodium pyruvate (1 mM), 10% heat-inactivated fetal bovine serum (FBS) and 50 ng/mL human GM-CSF (Novoprotein, CC79) for 7 days.

The Phorbol 12-myristate 13-acetate (PMA)-treated human macrophage cell line THP-1 and hMDMs was infected with BCG at a multiplicity of infection (MOI) of 10 bacilli per 1 cell for 6 h to serve as the target cells. After infection, extracellular non-internalized bacilli were removed by washing with pre-warmed PBS. For the intracellular mycobacterial growth inhibition assay, mycobacteria-infected THP-1 macrophages or hMDMs cells were co-cultured with purified CD3^+^, IFNγ^+^CD3^+^ and IFNγ^-^CD3^+^ cells, respectively, at a ratio of 1:10 for three days. At last, the infected cells were lysed in sterile PBS with 0.067% SDS. A serial dilution was performed for quantitative culturing on Middlebrook 7H10 agar (Becton Dickinson, 262710) plates supplied with 10% OADC for 2–3 weeks until colonies were large enough to be counted (26).

### 2.4 Transcriptome profiling of IFNγ^+^CD3^+^ and IFNγ^-^CD3^+^ cells

IFNγ^+^CD3^+^ and IFNγ^-^CD3^+^cells were obtained from the different treatment groups and subjected to mRNA-seq for transcriptome profiling. Total RNA was extracted from the cells using commercially available kits (Zymo Research, R2062). A poly-A mRNA library was constructed using the Illumina TruSeq mRNA Library Prep Kit, and the sequencing was performed on the Illumina NovaSeq 6000 platform.

### 2.5 Differential expression analysis

The quality of strand-specific paired-end RNA-seq reads was first assessed using FastQC (https://www.bioinformatics.babraham.ac.uk/projects/fastqc/) and then trimmed for adaptor sequences and low-quality bases using Fastp (27). The reads were aligned to the genome using Hisat2 (28). The gene-level expression values were obtained counts using htseq-count (29). Differentially expressed genes (DEG) between groups were identified using the DESeq2 R package v1.36.0 in R with P < 0.05, and |log_2_FC|≥1.0 as the thresholds (30). The ggplot2 R package (cran.r-project.org) was used to plot volcano plots. A heat map was generated using ComplexHeatmap (31).

For differential isoform expression analysis, the genome assembly and annotation were downloaded from GENCODE (version 46) (https://www.gencodegenes.org/human/). The reads were mapped to the transcriptome using STAR (32), and isoform-level expression values were quantified using RSEM (33). The DESeq2 R package was used to estimate the statistical significance of differentially expressed isoforms, which were identified with P < 0.05, and |log_2_FC|≥1.0.

BCG and Mtb IFNγ^+^CD3^+^ signature genes were defined as upregulated genes in BCG or Mtb IFNγ^+^CD3^+^ cells compared with the corresponding IFNγ^-^CD3^+^ cells. Genes concatenated between BCG and Mtb IFNγ^-^CD3^+^ signature genes were regarded as IFNγ^-^CD3^+^ signature genes. Signature isoforms with a mean transcript per million (TPM) > 1 in at least one of the three groups were obtained using a similar method as signature genes, except that the differentially expressed isoforms at the isoform level rather than the gene level were retained. The R package “ggtern” (version 3.3.0) was used to draw ternary phase diagrams.

### 2.6 Functional enrichment analysis

Using an adjusted P value cutoff of 0.05, and a |log_2_FC|≥1.0, we determined the set of genes that were significantly upregulated or downregulated. Reactome pathway enrichment associated with DEG in each cell type was identified using the *enrichment pathway* function in the ReactomePA package v1.40.0 in R (34). We considered reactome pathway enrichment to be statistically significant if the Benjamini-Hochberg test adjusted P < 0.05.

The Metascape online server (35) was used to carry out GO and pathway enrichment analysis with the “Custom Analysis” option. The top 10 enriched terms were selected for network visualization. Cytoscape (version 3.9.1) (36) was used to modify and visualize the network of the enriched terms. To perform enrichment analysis of isoforms with protein-coding capabilities, we initially mapped Ensembl isoform IDs to Ensembl protein IDs, followed by inputting them into Metascape.

### 2.7 Analysis of immune cell type abundance

Digital cytometry was performed using the online tool CIBERSORTx as previously described (37). Briefly, we obtained a given feature immune gene expression set from CIBERSORT (38), namely LM22.txt, and then categorized the different immune cell subtypes of IFNγ^+^CD3^+^ and IFNγ^-^CD3^+^ samples according to all genes detected.

### 2.8 Quantification of gene expression by qRT-PCR

RNA from enriched and stimulated IFNγ^+^CD3^+^ and IFNγ^-^CD3^+^ cells was reverse transcribed (TAKARA, RR047A) and amplified by qRT-PCR (TAKARA, RR820Q). The primers used for amplification are listed in **Table 1**. β-actin was used as a reference gene for normalization. Relative expression was calculated using the ΔΔCT method.

**Table 1:** Oligonucleotide sequences of the qRT-PCR primers (39).

Primers name	Forward primer	Reverse primer	Sequence Length (bp)
IFNG	TCGGTAACTGACTTGAATGTCCA	TCGCTTCCCTGTTTTAGCTGC	93
IL2	ACCCAGGGACTTAATCAGCAA	TGCTGTCTCATCAGCATATTCAC	94
TNFRSF9	AGCTGTTACAACATAGTAGCCAC	GGACAGGGACTGCAAATCTGAT	137
GZMB	CCACTCTCGACCCTACATGG	GGCCCCCAAAGTGACATTTATT	141
TNFA	CCTCTCTCTAATCAGCCCTCTG	GAGGACCTGGGAGTAGATGAG	119
CCL4	CTCCTCATGCTAGTAGCTGCCTTC	GGTGTAAGAAAAGCAGCAGGCGGT	109
β-ACTIN	CGAGAAGATGACCCAGAT	GATAGCACAGCCTGGATA	75

### 2.9 Flow cytometric analysis

This procedure was performed as described previously (24, 25). For surface molecules staining, cells were stained with the following antibodies: live-dead (Zombie NIR, Biolegend), anti-hu-CD3 (Clone SP34-2, BD), anti-hu-CD107a (Clone H4A3, Biolegend), anti-hu-CD137 (Clone 4B4-1, Biolegend), For intracellular molecule staining, cells were fixed, permeabilized, and stained with anti-hu-IFNγ (Clone 4SB3, BioLegend), anti-hu-TNFα (Clone MAb11, BioLegend), anti-hu-GZMB (Clone GB11, BioLegend), and anti-hu-IL-2 (Clone MQ1-17H12, BioLegend) antibodies or relevant isotype antibodies, as described previously (21, 40).

After staining, cells were fixed with 2% formaldehyde-PBS (Protocol Formalin, Kalamazoo, MI) and subjected to run on CYTOFLEX flow cytometer (Beckman). Lymphocytes were gated based on forward and side scatter, and at least 40,000 gated events were analyzed using CytExpert data acquisition and analysis software (Beckman).

### 2.10 Statistical analysis

Results from qRT-PCR and flow cytometric analyses were performed using GraphPad Prism 9.0. Differences between groups were assessed using t-test or nonparametric test, followed by Dunnett’s test or Tukey’s multiple comparison test, as indicated in the figure.

## 3 Results

### 3.1 IFNγ-secreting T cells induced by Mtb antigen could more potently inhibit mycobacteria growth in macrophages than IFNγ^-^CD3^+^ T cells

**Figure 1:**
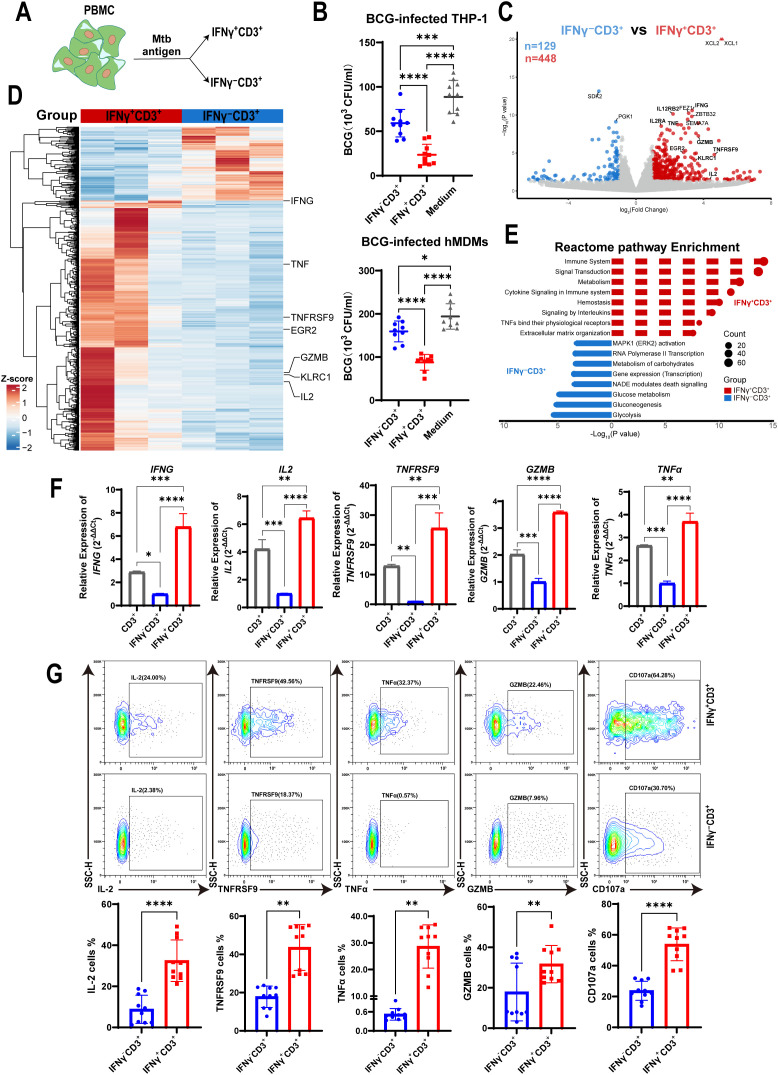
IFNγ^+^CD3^+^ cells more potently inhibited intracellular mycobacterial growth in macrophages with a high expression of effector cytokines. **(A)** Schematic showed fresh PBMCs were stimulated with the Mtb antigen for 6 h, and then were subjected to isolation of IFNγ^+^CD3^+^ (IFNγ-producing T cells) and IFNγ^-^CD3^+^ cells. **(B)** BCG-infected THP-1 macrophages or hMDMs as target cells, co-cultured with IFNγ^-^CD3^+^ cells, IFNγ^+^CD3^+^ cells,and medium (as control) for 3 days, and BCG CFU were counted (N=9, 10). **(C)** Volcano plots showed the difference of gene expression profiles between IFNγ^+^CD3^+^ (red) and IFNγ^-^CD3^+^ (blue) cells after Mtb antigen stimulation. A total of 448 genes were significantly upregulated in IFNγ^+^CD3^+^ T cells, and 129 genes were significantly upregulated in IFNγ^-^CD3^+^ T cells. **(D)** Heat map showed the clustering results of differentially expressed genes among the IFNγ^+^CD3^+^ vs IFNγ^-^CD3^+^ comparisons. Clustering was performed using the average linkage and Pearson’s correlations. Values are shown in terms of z-scores scaled by each gene. **(E)** Reactome pathway-enriched categories between IFNγ^+^CD3^+^ (red) and IFNγ^-^CD3^+^ (blue) T cells. **(F)** The expression levels of *IFNG*, *IL2*, *TNFRSF9*, *GZMB*, *TNFα* were determined by q-PCR in total CD3(gray), IFNγ^+^CD3^+^ (red) and IFNγ^-^CD3^+^ (blue) cells stimulated by Mtb antigen *in vitro* (N=3). **(G)** Representative flow cytometry histograms **(G)** and dot graphs **(H)** showed the frequencies of IL-2, TNFRSF9, TNFα, GZMB, and CD107a-producing cells among IFNγ^+^CD3^+^ T cells and IFNγ^-^CD3^+^ T cells (N=10), respectively. Results are expressed as mean ± SD. *p < 0.05, **p < 0.01, ***p < 0.001, ****p < 0.0001. Statistical significance was determined using one-way ANOVA **(B, F)**, or Student’s t-test **(G)**. Data represent 3 independent experiments.

To evaluate whether IFNγ-producing T cells stimulated by Mtb antigen can regulate intracellular Mtb growth in macrophages, we stimulated PBMCs from healthy donors with the antigen (HMBPP and PPD). This allowed us to isolate IFNγ^+^CD3^+^ and IFNγ^-^CD3^+^ T cells, respectively (**Figure 1A**). These purified T cells were then co-cultured with BCG-infected THP-1 macrophages for three days. After incubation, the cells were lysed and plated on 7H10 agar medium to count BCG colony-forming units (CFU) as described previously (25). Our results showed that the mean CFU count of BCG in the IFNγ^-^CD3^+^ T cell co-culture was significantly lower than in the control group of BCG-infected THP-1 macrophages (59.07×10³ CFU/ml vs. 88.67×10³ CFU/ml). More notably, the average BCG CFU count in the IFNγ^+^CD3^+^ T cell co-culture was approximately half that of the IFNγ^-^CD3^+^ T cell treatment (23.53×10³ CFU/ml vs. 59.07×10³ CFU/ml). Next, we co-cultured IFNγ^+^CD3^+^ T cells with hMDMs infected with BCG. Consistently, we found that the mean CFU count of BCG in the IFNγ^+^CD3^+^ T cell-treated Mφ co-culture was also significantly lower than those of the other co-cultures of IFNγ^-^CD3^+^ T cells and medium control, respectively (**Figure 1B**). These findings indicate that IFNγ-producing (IFNγ^+^CD3^+^) T cells have a greater ability to suppress intracellular mycobacterial growth in macrophages compared to their counterparts of IFNγ^-^CD3^+^ T cells. Although IFNγ^-^CD3^+^ T cells can significantly inhibit mycobacterial growth, IFNγ^+^CD3^+^ T cells are markedly more effective (**Figure 1B**).

To identify why IFNγ-producing T cells displayed a more potent inhibitory effect on intracellular mycobacteria growth, we stimulated PBMCs with antigen and isolated IFNγ^+^CD3^+^ and their counterparts of IFNγ^-^CD3^+^ T cells for mRNA sequencing. Sequencing data analysis showed that 448 genes were upregulated in IFNγ^+^CD3^+^ T cells (P < 0.05, |log_2_FC|≥1.0) compared with their counterparts in IFNγ^-^CD3^+^ T cells, whereas 129 genes were upregulated in IFNγ^-^CD3^+^ T cells (**Figure 1C**). Many effector molecules were upregulated in IFNγ^+^CD3^+^ T cells, including *IFNG*, *TNF*, *GZMB*, *TNFRSF9*, and *IL2* (**Figures 1C, D**), all of which played crucial roles in the immune response against Mtb infection.

Pathway enrichment analysis revealed that many upregulated genes in IFNγ^+^CD3^+^ T cells were enriched in several categories (P < 0.05), including the immune system, signal transduction, cytokine signaling in the immune system, signaling by interleukins, and TNFs binding their receptors. Together, these pathways are essential for enabling the host immune system to respond rapidly and accurately to external pathogens (41–43) (**Figure 1E**, **Supplementary Table 1**). In contrast, genes upregulated in IFNγ^-^CD3^+^ T cells showed different sets of enriched categories (adjusted P < 0.05), including MAPK1 activation, RNA polymerase II transcription, and carbohydrate metabolism, which play critical role in coordinating cell proliferation, gene expression, and energy metabolism (44, 45) (**Figure 1E**, **Supplementary Table 1**).

To confirm the expression of effector cytokines identified in mRNA sequencing data, we detected the relative expression of *IFNG*, *TNF*, *GZMB*, *TNFRSF9*, and *IL2* genes by qRT-PCR in IFNγ^+^CD3^+^ T cells compared to IFNγ^-^CD3^+^ T cells. The results showed that the relative expression of *IFNG*, *TNF*, *GZMB*, *TNFRSF9*, and *IL2* genes in IFNγ^+^CD3^+^ T cells was approximately 4.7-, 5-, 24-, 2.4-, and 1.7-fold, respectively, of that in IFNγ^-^CD3^+^ T cells (**Figure 1F**). Our flow cytometry results added to a growing body of evidence that the frequencies of these cytokine-producing cells were significantly enhanced in IFNγ^+^CD3^+^ cells compared to IFNγ^-^CD3^+^ T cells (**Figure 1G**).

To identify whether IFNγ^+^CD3^+^ T cells could kill mycobacteria in macrophages through the immune secretion of lytic granules, we detected the expression of CD107a in IFNγ^+^CD3^+^ and IFNγ^-^CD3^+^ T cells by flow cytometry. The results showed that there were significantly higher frequencies of CD107a positive cells in IFNγ^+^CD3^+^ T cells induced by antigens than in IFNγ^-^CD3^+^ T cells (**Figure 1G**). These results indicate that the main mechanism by which IFNγ^+^CD3^+^ T cells control intracellular mycobacterial growth might be related to degranulation, as indicated by the surface expression of CD107a, in a manner akin to that of CTL and NK cells (46).

### 3.2 Gene expression patterns of IFNγ^+^CD3^+^ cells induced by BCG were different from those induced by Mtb

**Figure 2:**
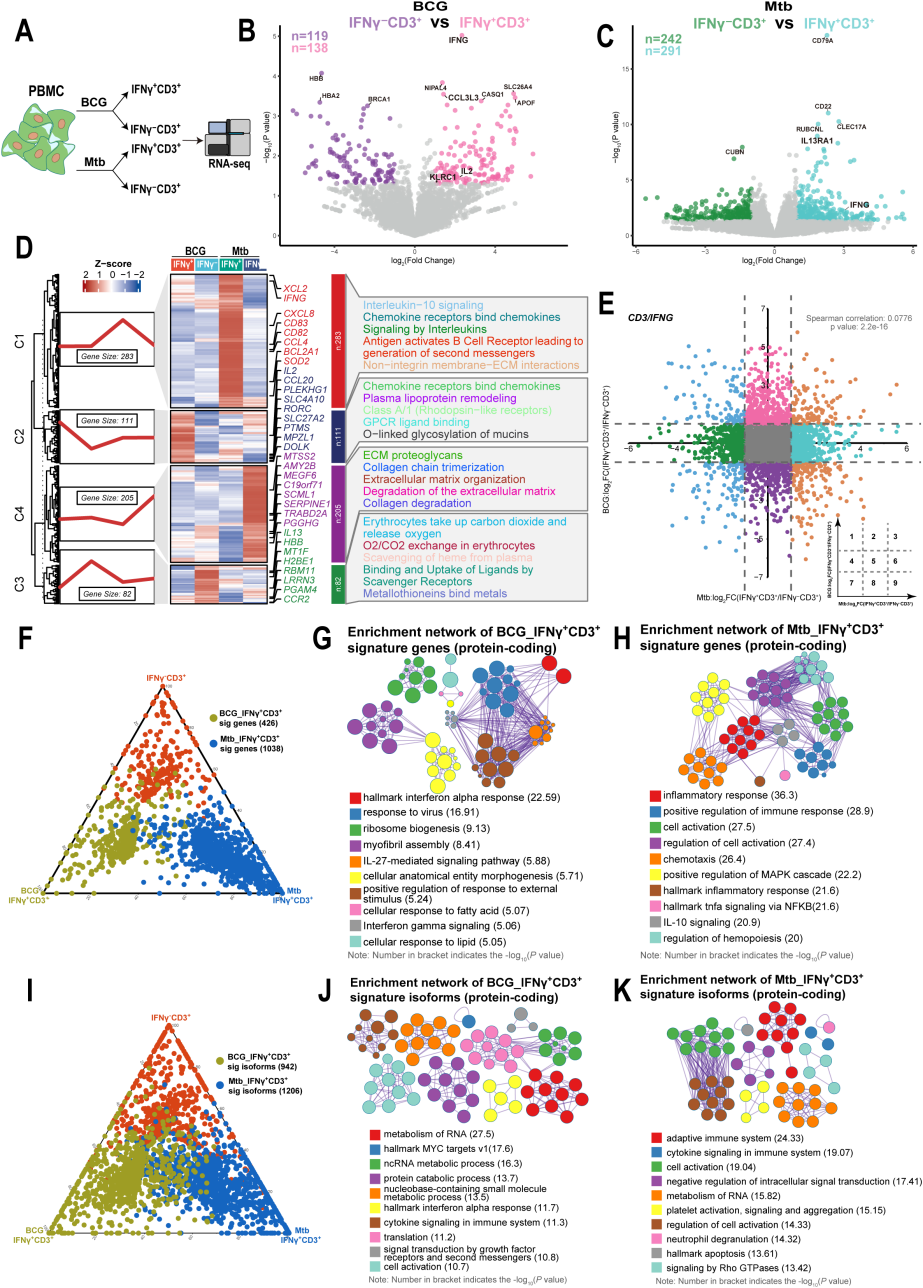
IFNγ^+^CD3^+^ cells induced by BCG showed different gene expression patterns with those induced by Mtb. **(A)** Schematic of isolated IFNγ^+^CD3^+^, IFNγ^-^CD3^+^ cells experimental design. Fresh PBMC were stimulated with BCG or Mtb for 6 hours. The cells were then subjected to phenotypic analysis and RNA-seq. **(B)** Volcano plot comparing IFNγ^+^CD3^+^ (pink) to IFNγ^-^CD3^+^ (purple) treated with BCG. The expression of 138 genes was significantly higher in IFNγ^+^CD3^+^ T cells, and that of 119 genes was significantly higher in IFNγ^-^CD3^+^ T cells. **(C)** Volcano plot comparing IFNγ^+^CD3^+^ (light blue) and IFNγ^-^CD3^+^ (green) cells treated with H37Rv. The expression of 291 genes was significantly higher in IFNγ^+^CD3^+^ T cells, and that of 242 genes was significantly higher in IFNγ^-^CD3^+^ T cells. **(D)** Gene expression patterns analysis. Left: 681 DEGs between IFNγ^+^CD3^+^ and IFNγ^-^CD3^+^ cells stimulated by BCG or Mtb categorized into four clusters based on their characteristic expression patterns. Middle: Heatmap showing the expression patterns of key dynamically expressed genes (681 genes). Right: Reactome pathways enriched in each gene cluster were identified. **(E)** The nine-quadrant diagram displays the expression patterns of genes across subgroups. All genes with |log_2_FC|≥1.0 are marked. In quadrant 1, genes are upregulated in BCG but downregulated in Mtb. Quadrant 9 shows genes upregulated in Mtb but downregulated in BCG. Quadrant 3 includes genes upregulated in both BCG and Mtb, while quadrant 7 shows genes downregulated in both. No significant correlations were found in quadrants 2, 4, 5, 6, and 8. **(F, I)** The ternary phase diagrams illustrate the relative enrichment of IFNγ^+^CD3^+^ T cells exposed to BCG or Mtb, focusing on signature genes **(F)** and protein-coding isoforms **(I)**. Signature isoforms were identified as those showing differences at the isoform level but not at the gene level. **(G, H)** The enrichment networks display the top 10 enriched terms for IFNγ^+^CD3^+^ T cells exposed to BCG **(G)** or Mtb **(H)** based on signature genes. **(J, K)** Similarly, enrichment networks represent the top 10 enriched terms for IFNγ^+^CD3^+^ T cells exposed to BCG **(J)** or Mtb **(K)** based on protein-coding signature isoforms. Enriched terms with high similarity were grouped into clusters and visualized as a network. Each node represents an enriched term, color-coded by its cluster. Node size corresponds to the number of enriched genes, and line thickness reflects the similarity score between terms. The term with the lowest P value in each cluster is labeled.

Previous research has shown that BCG vaccination programs induce HSCs in the bone marrow through the IFN-II response, which provides trained immunity against Mtb infection. However, Mtb infection reprograms HSCs through the IFN-I pathway, which impairs the development of protective immunity (20). Here, we focused on isolating IFNγ^+^CD3^+^ T cells from PBMCs of human subjects following stimulation with BCG and Mtb. We then determined the gene expression profiles of IFNγ^+^CD3^+^ T cells and their corresponding IFNγ^-^CD3^+^ T cells (**Figure 2A**). Our findings revealed that 138 genes were upregulated and 119 genes were downregulated in the IFNγ^+^CD3^+^ T cells induced by BCG compared to the IFNγ^-^CD3^+^ T cells (**Figure 2B**). In addition, 291 genes were upregulated and 242 genes were downregulated in Mtb-induced IFNγ^+^CD3^+^ T cells compared to their counterparts in IFNγ^-^CD3^+^ T cells (**Figure 2C**).

To further characterize gene expression profiles, we clustered differentially expressed genes between IFNγ^+^CD3^+^ and IFNγ^-^CD3^+^ cells stimulated by BCG or Mtb and performed a reactome pathway analysis. We found that 681 differentially expressed genes were categorized into four clusters based on their characteristic expression patterns. Among them, 283 genes in cluster C1 were highly expressed in IFNγ^+^CD3^+^ T cells induced by Mtb, and these genes were mainly enriched in interleukin-10 signaling, chemokine receptor-binding chemokines, signaling by interleukins, and antigen-activating B cell receptors, leading to the generation of second messengers and non-integrin membrane-ECM interactions (**Figure 2D**, **Supplementary Table 2**). Similarly, 111 genes in cluster C2, mainly upregulated in IFNγ^+^CD3^+^ T cells induced by BCG, were enriched in chemokine receptors that bind chemokines, plasma lipoprotein remodeling, classA/1 (Rhodopsin-like receptors), GPCR ligand binding, and O-linked glycosylation of mucines (**Figure 2D**, **Supplementary Table 2**). These results indicated that there were different gene expression patterns in IFNγ^+^CD3^+^ T cells induced by Mtb compared to those induced by BCG.

To investigate the correlation of gene expression between IFNγ^+^CD3^+^ T cells induced by BCG and those induced by Mtb, we analyzed the changes in gene expression levels in IFNγ^+^CD3^+^ T cells compared with IFNγ^-^CD3^+^ T cells after stimulation with BCG and Mtb. Nine-quadrant diagram analysis showed that 95, 335, 116, 494, 12220, 486, 110, 334, and 158 genes were distributed in quadrants 1-9, respectively (**Figure 2E**). Further analysis revealed no correlation between changes in gene expression levels in BCG-stimulated IFNγ^+^CD3^+^ T cells and Mtb-stimulated cells (Spearman correlation = 0.0776). This suggested that although BCG and Mtb shared approximately 98% of genomes (47, 48), they presented different immune effect on stimulation of IFNγ^+^CD3^+^ T cells, and it might be consistent with their different clinical outcomes.

As traditional transcriptional analyses consider all isoforms of an expressed gene together, despite potential functional differences (49). Furthermore, we comprehensively assessed the differences in gene expression and transcriptional isoform signatures between BCG and Mtb IFNγ^+^CD3^+^ T cells. We identified genes that were highly expressed in either BCG or Mtb IFNγ^+^CD3^+^ T cells and compared their transcriptional profiles (**Figure 2F**). Our results showed that the functions of highly expressed genes in BCG IFNγ^+^CD3^+^ T cells highly expressed genes were mainly enriched in terms related to interferon signaling and ribosome biogenesis (**Figure 2G**), whereas Mtb IFNγ^+^CD3^+^ T cells were mainly associated with inflammatory response and cell activation (**Figure 2H**). These findings suggested that BCG and Mtb IFNγ^+^CD3^+^ T cells had distinct functional characteristics despite of same genome.

We also identified isoforms that were notably more highly expressed in BCG and Mtb IFNγ^+^CD3^+^ T cells than in the corresponding IFNγ^-^CD3^+^ cells (**Figure 2I**). BCG IFNγ^+^CD3^+^ T cells exhibited significant enrichment of protein-coding isoforms in various metabolic processes (**Figure 2J**), whereas Mtb IFNγ^+^CD3^+^ T cells displayed predominant enrichment of protein-coding isoforms in immune-related terms (**Figure 2K**). The enrichment of signature protein-coding isoforms in RNA metabolic processes in both BCG and Mtb IFNγ^+^CD3^+^ T cells support the hypothesis of the global activation of the post-transcriptional program in the peripheral immune system in response to BCG and Mtb stimulation, particularly in BCG IFNγ^+^CD3^+^ T cells.

### 3.3 IL-2 was highly expressed in IFNγ^+^CD3^+^ T cells induced by Mtb antigen and BCG but not Mtb bacteria

**Figure 3:**
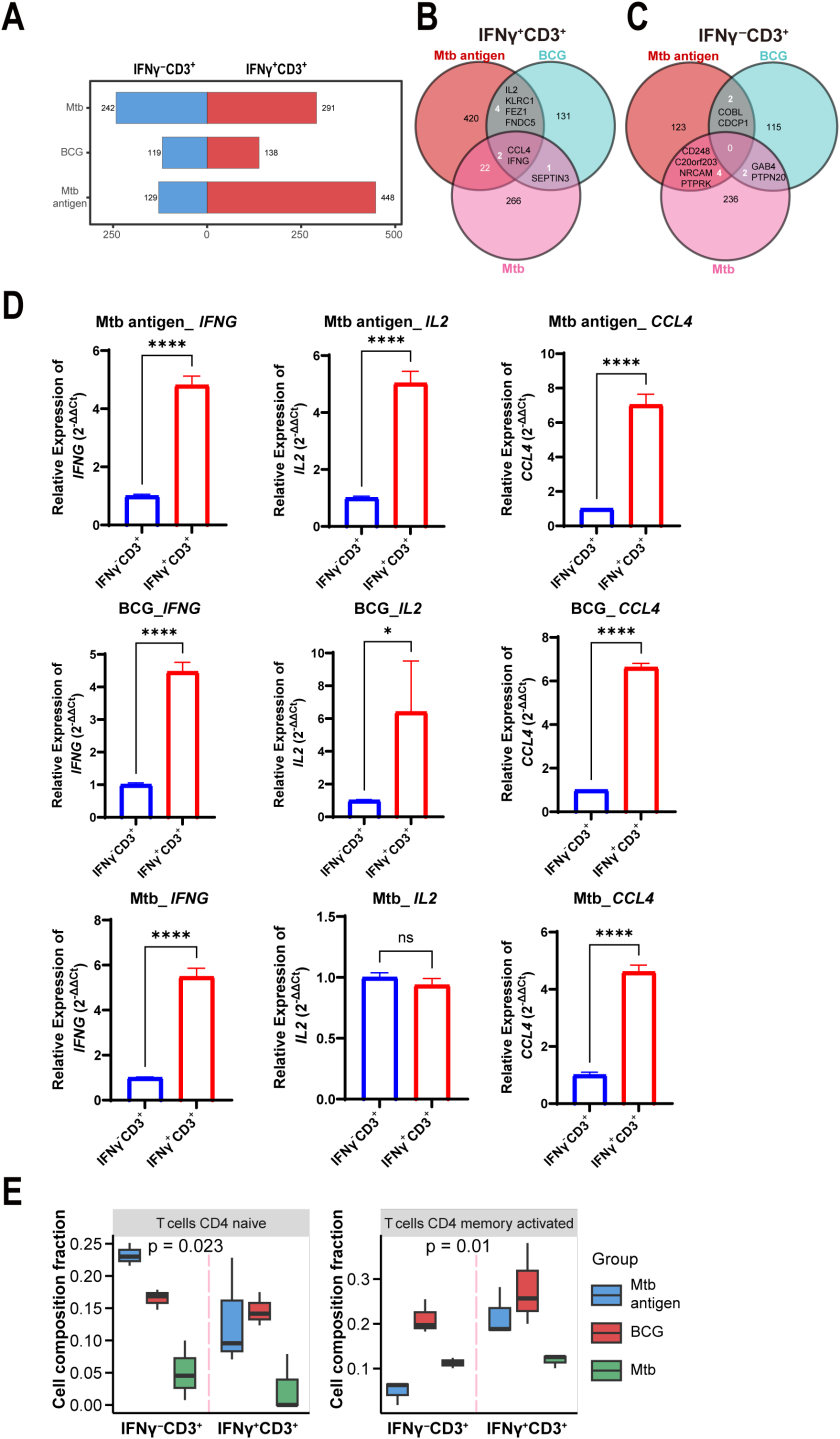
IL-2 was highly expressed in IFNγ^+^CD3^+^ T cells induced by antigen and BCG, but not Mtb. **(A)** Graph demonstrates all number of DEG among the IFNγ^+^CD3^+^ vs IFNγ^-^CD3^+^ comparisons stimulated by antigen, BCG and Mtb. **(B, C)** Venn diagram showing overlapping DEGs in IFNγ^+^CD3^+^ cells **(B)** and IFNγ^-^CD3^+^ cells **(C)** stimulated by antigen, BCG, and Mtb. **(D)** *CCL4*, *IFNG*, and *IL2* expression levels were determined by qRT-PCR in IFNγ^+^CD3^+^ cells and IFNγ^-^CD3^+^ cells stimulated by antigen, BCG, and Mtb, respectively (N=3). **(E)** Graph showed the frequencies of the CD4 naive T cells, CD4 memory activated T cells, and CD4 memory resting subpopulations in IFNγ^+^CD3^+^ cells and IFNγ^-^CD3^+^ cells stimulated by antigen, BCG, Mtb, respectively. Results are expressed as mean ± SD. ns, not significant, *p < 0.05, ****p < 0.0001. Statistical significance was determined using Student’s t-test. Data represent 3 independent experiments.

To further characterize the stimulus-dependent priming of IFNγ^+^CD3^+^ T cells, we analyzed their gene expression profiles following exposure to Mtb antigen, BCG vaccine, and Mtb pathogen. The number of DEG between IFNγ^+^CD3^+^ and IFNγ^-^CD3^+^ cells differed among the three types of stimulation (**Figure 3A**). A total of 291, 138, and 448 genes were upregulated, and 242, 119, and 129 genes were downregulated in IFNγ^+^CD3^+^ T cells induced by Mtb, BCG, and antigen, respectively, compared to their counterparts in IFNγ^-^CD3^+^ cells (**Figure 3A**). As depicted in the figure, BCG stimulation resulted in a lower number of DEG and a lower fold change of gene expression compared to other stimuli (**Figure 3A**). Seemingly, BCG would induce more moderate immune response comparing to Mtb antigen and Mtb bacteria.

Among upregulated genes in IFNγ^+^CD3^+^ T cells, two genes of *IFNG* and *CCL4* co- expressed in all three cell subtypes (**Figure 3B**). In addition, there were four other common genes, *IL2*, *KLRC1*, *FEZ1*, and *FNDC5*, between antigen and BCG-induced IFNγ^+^CD3^+^ T cells (**Figure 3B**). However, there were no common genes in IFNγ^-^CD3^+^ T cells (**Figure 3C**), indicating the absence of a shared gene signature.

To further confirm the expression of these genes, we tested their expression levels with qRT-PCR methods in more examples. Results showed that all the expression levels of *IFNG* increased more than 4 times in IFNγ^+^CD3^+^ T cells comparing to IFNγ^-^CD3^+^ T cells treated with antigen, BCG, and Mtb, respectively (**Figure 3D**). Similarly, *CCL4* was also upregulated in all IFNγ^+^CD3^+^ T cells (**Figure 3D**). Notably, qRT-PCR results also verified that *IL2* was significantly enhanced in IFNγ^+^CD3^+^ T cells compared to IFNγ^-^CD3^+^ T cells treated with antigen and BCG, but not Mtb (**Figure 3D**).

Consistent with the findings of RNA-seq analysis, qRT-PCR analysis also demonstrated that *IFNG* and *CCL4* were highly expressed in IFNγ^+^CD3^+^ T cells compared to IFNγ^-^CD3^+^ T cells treated with antigen, BCG, and Mtb (**Figure 3D**). However, *IL2* was highly expressed in IFNγ^+^CD3^+^ T cells compared to IFNγ^-^CD3^+^ T cells treated with antigen and BCG, but not Mtb (**Figure 3D**). Our and other studies have shown that *IL2* plays an important role in anti-Mtb infection, significantly reducing the bacterial burden in the lungs of multidrug-resistant tuberculosis (MDR-TB)-infected hosts, resulting in milder pathology/lesions and improved treatment outcomes (50).

Furthermore, to assess the cell subset composition, we analyzed RNA sequencing data of IFNγ^+^CD3^+^ and IFNγ^-^CD3^+^ T cells treated with antigen, BCG, and Mtb using the CIBERSORTx tool. Our results showed that there were lower levels of CD4 naïve T cells and CD4 memory activated T cells in IFNγ^+^CD3^+^ T cells induced by Mtb than in those induced by BCG and antigen (**Figure 3E**), These might indicate that antigen and BCG would induce more protective immune response than Mtb pathogen, since immune cell types and their quantities have been linked to TB outcomes, with differing infiltration of these cells in the lung microenvironment relating to Mtb survival (51–54).

### 3.4 IL-2 helps BCG potently induce more effector IFNγ^+^CD3^+^ T cells

**Figure 4:**
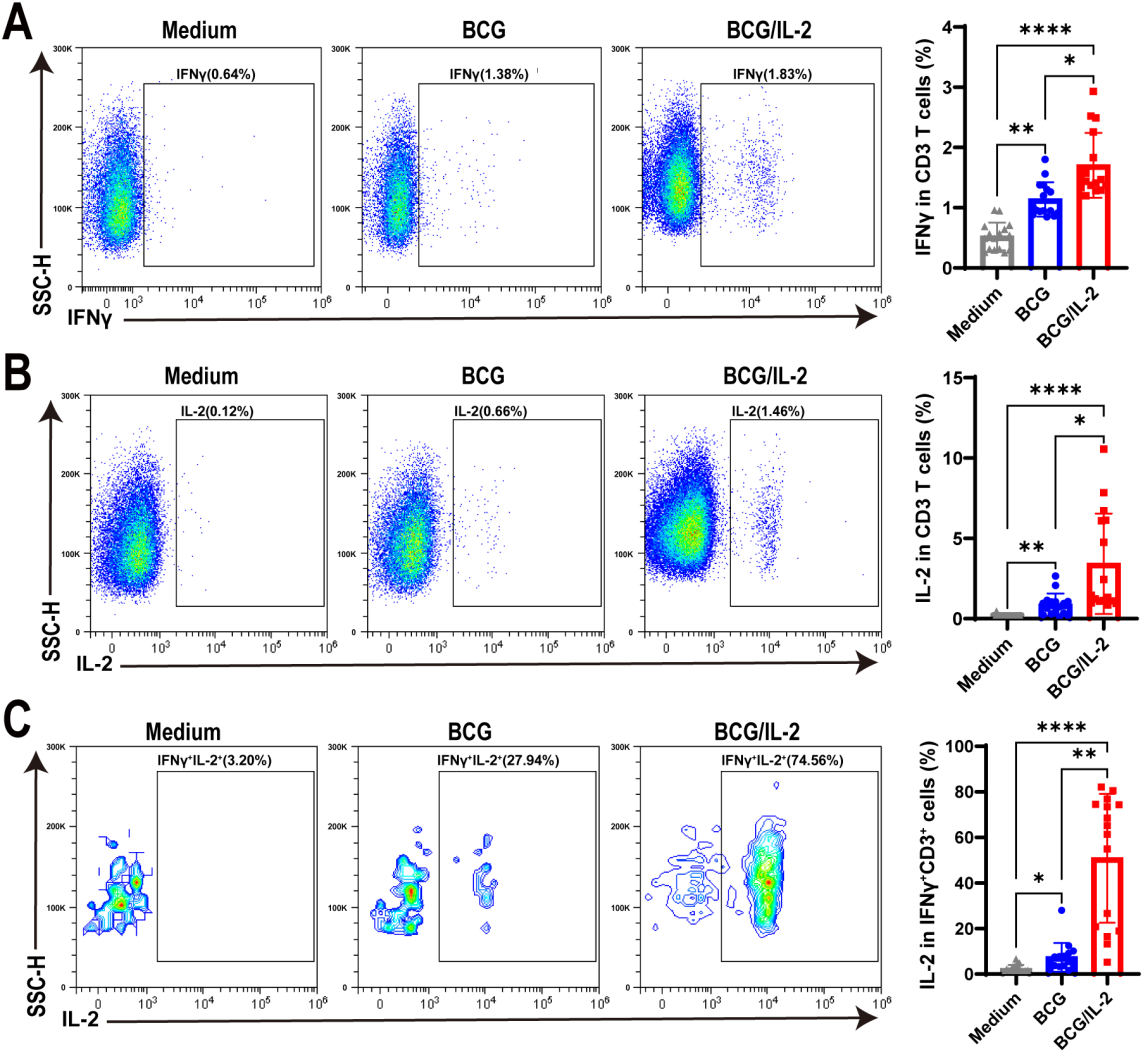
IL-2 treatment remarkably activated IFNγ^+^CD3^+^ T cells in HC PBMC. **(A–C)** The percentages of IFNγ^+^ **(A)** IL-2^+^ **(B)** and IFNγ^+^IL-2^+^ **(C)** cells stimulated by BCG, or BCG plus IL-2, respectively, were analyzed by flow cytometry (N=17). Results are expressed as mean ± SD. *p < 0.05, **p < 0.01, ****p < 0.0001. Statistical significance was determined using one-way ANOVA. Data represent 3 independent experiments.

As our above results concluded that BCG and antigen, not Mtb bacteria, induced IFNγ^+^CD3^+^ T cells to exhibit high levels of IL-2 expression, we hypothesized that IL-2 might be helpful in promoting differentiation of IFNγ^+^CD3^+^ T cells. To address this, we treated PBMC cells with BCG, BCG plus IL-2 and medium control, and found that the frequencies of IFNγ producing T cells in BCG plus IL-2 treatment were significantly higher than those treated by BCG, which also obviously increased IFNγ production in T cells comparing to medium control (**Figure 4A**). Similarly, we found that IL-2 plus BCG induced higher IL-2 production than BCG alone, with an average of 3.2% IL-2^+^ T cells (**Figure 4B**). Interestingly, our results also revealed that BCG plus IL-2 stimulation led to a considerable increase in the proportion of IFNγ^+^IL-2^+^ cells (mean 45.55%) compared to BCG and the medium control (**Figure 4C**). Overall, the above results demonstrated that IL-2 potently activated IFNγ^+^CD3^+^ T cells induced by BCG and increased effector cytokines production.

### 3.5 TB infection decreased IL-2 production in IFNγ^+^CD3^+^ T cells

**Figure 5:**
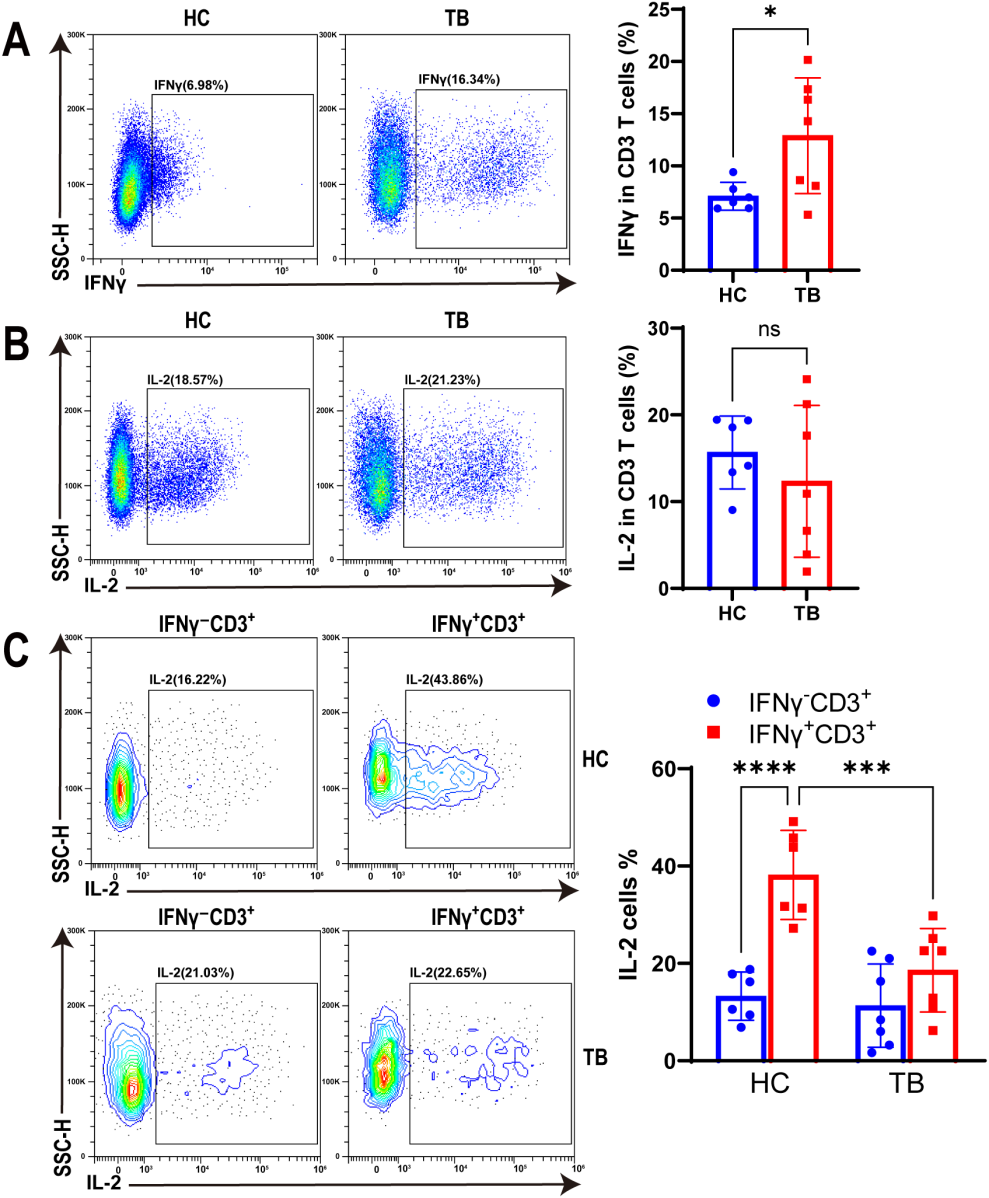
TB infection decreased IL-2 production in IFNγ^+^CD3^+^ T cells. **(A, B)** Representative flow cytometry histograms (left) and dot graphs (right) illustrated the frequencies of IFNγ^+^CD3^+^ cells **(A)** and IL-2 producing cells **(B)** from HC donors and TB patients with Mtb antigen stimulation, respectively (N=6,7). **(C)** The frequencies of IL-2 producing cells in IFNγ^+^CD3^+^ and IFNγ^-^CD3^+^ cells from HC donors and TB patients were determined by flow cytometry after Mtb antigen stimulation, respectively (N=6,7). Results are expressed as mean ± SD. *p < 0.05, ***p < 0.001, ****p < 0.0001. Statistical significance was determined using Student’s t-test **(A, B)**, one-way ANOVA **(C)**. Data represent 3 independent experiments.

To characterize the function of IFNγ^+^CD3^+^ T cells under TB infection, we tested IFNγ and IL-2 expression in IFNγ^+^CD3^+^ T cells of HC and TB patients with antigen stimulation using flow cytometry. Our results showed that IFNγ expression in CD3^+^ T cells of TB patients were significantly higher than HC subjects (**Figure 5A**). However, there was no significant difference in IL-2 expression levels in CD3^+^ T cells between the two cohorts (**Figure 5B**).

In addition, our findings revealed that the expression level of IL-2 in IFNγ^+^CD3^+^ T cells was significantly higher in the HC cohort than in IFNγ^-^CD3^+^ T cells (**Figure 5C**). However, there was no significant difference in the IL-2 expression levels of these two cell subtypes in TB patients (**Figure 5C**). Notably, the expression level of IL-2 in IFNγ^+^CD3^+^ T cells was significantly higher in the HC cohort than in TB patients (**Figure 5C**). This suggests that Mtb infection may have impacted the expression of IL-2 in IFNγ^+^CD3^+^ T cells, which could potentially compromise its anti-tuberculosis effector.

## 4 Discussion

IFNγ and its receptor (IFN-γR) are crucial for immunity against Mtb infection (55). While previous research has focused on the overall role of cytokine IFNγ in TB immunity, there is limited information on the precise function of T cells producing IFNγ in controlling intracellular bacterial growth. In this study, for the first time, we successfully isolated IFNγ-secreting T cells induced by Mtb antigen using surface staining methods instead of intracellular staining. These intact IFNγ-secreting T cells were then co-cultured with mycobacteria-infected macrophages. Our results showed that the antigen-induced IFNγ-secreting T cells significantly inhibited the growth of intracellular mycobacteria in macrophages.

In our assay, we used structurally and functionally intact cells for mRNA sequencing to characterize the gene expression profiles of IFNγ^+^CD3^+^ T cells. We observed that the gene expression profiles of IFNγ^+^CD3^+^ T cells varied under different stimulations, namely antigen, BCG, and Mtb. Notably, *IFNG* and *CCL4* were highly expressed in these cells. IFNG, a specific marker, was consistently elevated in IFNγ^+^CD3^+^ T cells. *CCL4*, a mitogen-inducible monokine with chemokinetic and inflammatory functions, also showed increased expression, paralleling *IFNG* under stimulation (56). However, the interaction between *IFNG* and *CCL4* warrants further investigation to understand their combined role in immune response.

Although both BCG and Mtb are live mycobacteria, and BCG is the only approved vaccine against Mtb infection, we found significant differences in the gene expression patterns of IFNγ^+^CD3^+^ T cells induced by BCG compared to those induced by Mtb. Interestingly, the gene expression profiles of IFNγ^+^CD3^+^ T cells stimulated by antigen, which included Mtb-secreted proteins and the phosphoantigen HMBPP, were more similar to those induced by BCG than by Mtb.

This difference may be because Mtb-induced immune responses help the bacteria evade host clearance (57), while antigen and BCG inoculation aid the host in controlling Mtb infection. It has been reported that BCG programs hematopoietic stem cells (HSCs) in the bone marrow via the IFNγ response, which confers protective trained immunity against Mtb infection. In contrast, Mtb reprograms HSCs through an IFN-I response, impairing the development of protective immunity (20). Since IFNγ is a crucial cytokine in the anti-Mtb immune response, IFNγ-secreting T cells are vital for early protective immune responses to Mtb infection.

Therefore, the immune responses of IFNγ^+^CD3^+^ T cells are closely related to the type of stimulus. In this study, we found that genes such as *IL2* were highly expressed in IFNγ^+^CD3^+^ T cells induced by BCG and antigen compared to IFNγ^-^CD3^+^ T cells. However, there was no significant difference in *IL2* expression between IFNγ^+^CD3^+^ and IFNγ^-^CD3^+^ T cells induced by Mtb. Since IL-2 is a critical cytokine for T cell differentiation through the regulation of STAT5 activation (58, 59), this suggests that Mtb infection may suppress IL-2 expression in IFNγ^+^CD3^+^ T cells, potentially helping the bacteria evade host immune clearance. Furthermore, we found that BCG plus IL-2 stimulation not only increased the frequencies of IFNγ^+^CD3^+^ T cells but also enhanced IFNγ and IL-2 production in these cells. This indicates that IL-2 is crucial for the protective immune response of IFNγ^+^CD3^+^ T cells.

In conclusion, our study demonstrates that IFNγ^+^CD3^+^ T cells effectively inhibit intracellular mycobacterial growth in macrophages. Unlike Mtb, BCG and antigen induce IFNγ^+^CD3^+^ T cells to express high levels of IL-2, which may be linked to their enhanced effector function. However, the more detailed mechanisms by which IFNγ^+^CD3^+^T cells inhibit the intracellular *Mtb* growth, and whether other T cell subsets are involved in this protective process are still unclear. Future studies are needed to address these issues.

## Data Availability

The raw sequence data reported in this paper have been deposited in the Genome Sequence Archive (Genomics, Proteomics & Bioinformatics 2021) in National Genomics Data Center (Nucleic Acids Res 2024), China National Center for Bioinformation/Beijing Institute of Genomics, Chinese Academy of Sciences (GSAHuman: HRA009087) that are publicly accessible at https://ngdc.cncb.ac.cn/gsa-human.
